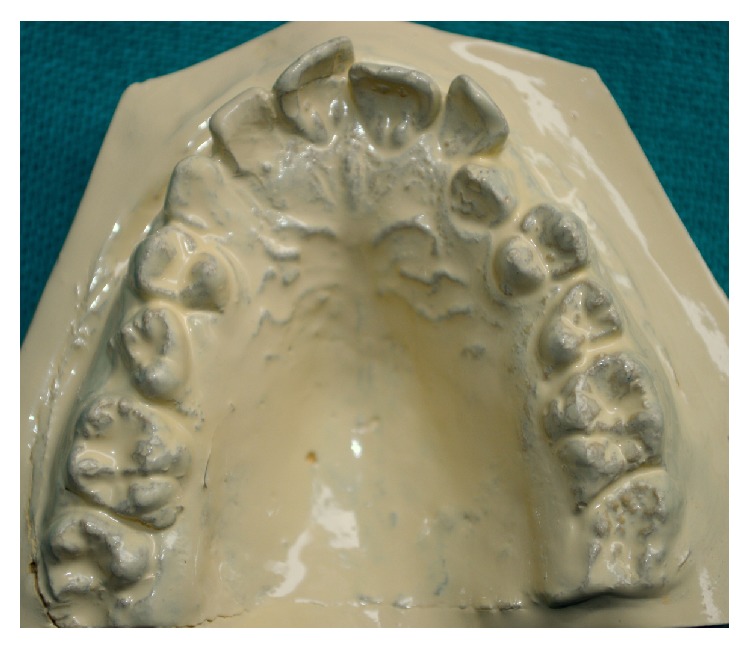# Bilateral Molariform Mandibular Second Premolars

**DOI:** 10.1155/2015/809463

**Published:** 2015-01-19

**Authors:** Sonu Acharya, Pradip Kumar Mandal, Chiranjit Ghosh

**Affiliations:** Department of Pedodontics and Preventive Dentistry, Institute of Dental Sciences, SOA University, Bhubaneswar, Odisha 751030, India

## Abstract

Macrodontia is a rare dental anomaly that refers to teeth that appear larger than normal. Generalised macrodontia can be associated with certain medical conditions and syndromes. This case report presents clinical and radiographic findings of isolated bilateral macrodontia in a 14-year-old child. The patient was referred to the clinic with local crowding of maxillary and mandibular teeth. Radiographic findings revealed the presence of impacted macrodont mandibular second premolar on one side and erupted macrodontic premolar on the other side and their distinct morphological appearance, characterized by large, multitubercular, and molariform crowns and tapering, single roots.

## 1. Introduction

Macrodontia is a morphoanatomical change that can affect any tooth in a way that the body of the tooth is enlarged and roots are smaller. The affected teeth have proportionately shortened roots, and their pulp chambers are enlarged as a result of apical prolongation. Teeth may occur in varying sizes and shapes that do not always correspond to the accepted descriptions. When dental size and anatomy present characteristics that deviate from what is supposed to be accepted range of normality, they are termed anomalies [1]. Mandibular second premolar has shown an elevated variability of crown morphology [2]. The anatomy of this tooth is particularly unpredictable, as are its eruptive potential and position in the dental arch [3]. The mandibular second premolar may present another extremely infrequent anomaly: molarization [4]. This molar-like morphology of the premolar consists of three buccal cusps and three, two, one, or no lingual cusps. The aetiology of dental anomalies remains largely unclear, but some anomalies in tooth structure, shape, and size result by many factors from disorders during the morphodifferentiation stage of development [5].

Identification of specific patterns of associated dental anomalies could be related to certain genetic and environmental factors contributing to different dental anomaly subphenotypes. Macrodontia (or megadontia) is a rare dental anomaly characterized by an excessive enlargement of all tooth structures and, in few cases, may be associated with morphological anomalies. Tooth anomaly can be categorized as follows: true generalized (large percentage of dentition), relative generalized (entire dentition), and isolated macrodontia of single tooth [6]. Multiple macrodontia is strange, but it may be associated with some diseases like insulin-resistant diabetes, otodental syndrome, or facial hemihyperplasia. Also, generalized macrodontia may be produced by hormonal imbalance, as has been described in pituitary gigantism. Macrodontia of a single tooth is a relatively uncommon condition and frequently has been reported in mandibular molars or premolars. It may affect incisors, third molars, and second mandibular premolars. It is characterized by excessive enlargement of mesiodistal and faciolingual tooth dimensions with an occlusal crown area increased [7]. The prevalence of macrodontia is 1-2% in males and 0.9% in females, but macrodontia of mandibular second premolars affects both sexes equally [8]. It is important to know macrodontia because it may cause problems with aesthetics and also with crowding if there is a discrepancy between the dimensions of the teeth and the size of the dental bases. Also, these teeth are more predisposed to caries and related to disruption of the developing occlusion by occlusal morphology.

## 2. Case Report

**Figure 1:**
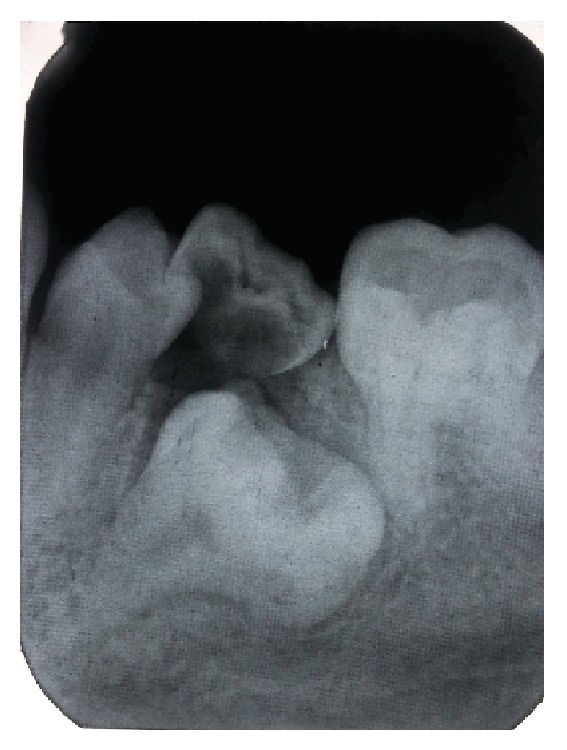


**Figure 2:**
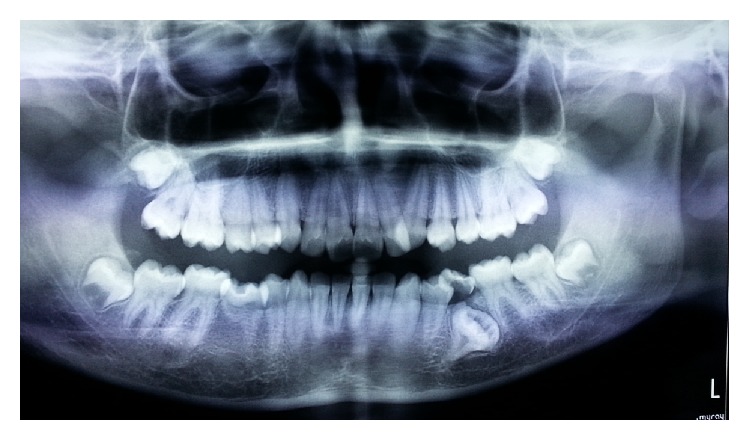


**Figure 3:**
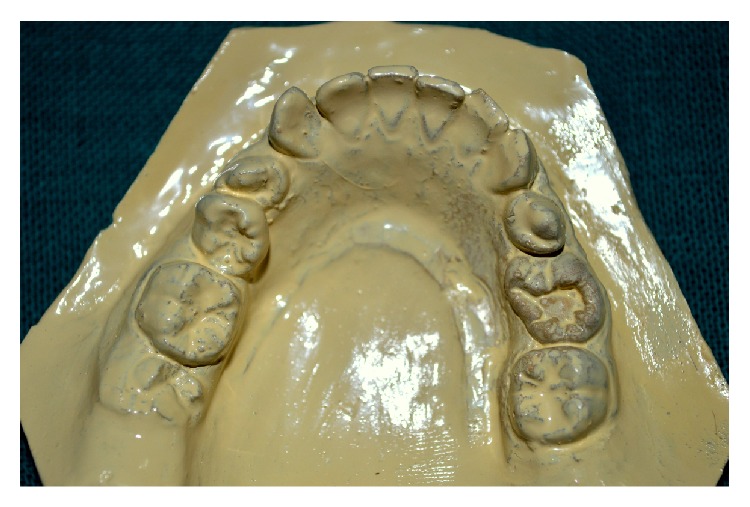


A 14-year-old female child visited the outpatient department of pediatric dentistry with a chief complaint of irregular teeth in oral cavity. The patient was nonsyndromic and all her vital signs were well within normal range. There was no familial history of any anomaly. On intraoral examination the patient had crowding in the mandibular arch and there was an unusually bulbous second premolar on the right side which had molar-like appearance. Intraoral periapical radiograph of the tooth showed that it had an enlarged pulp chamber and short roots, suggestive of macrodontia (Figure 1). On the left side second deciduous molar was retained. Orthopantomograph revealed impacted second premolar on the left side which was also quite bulbous with large crown and pulp chamber with comparatively smaller roots (Figure 2). Both the premolars had multiple cusps leading to suggestion of being molariform in nature as described in literature. No other obvious dental anomalies were noticed on the orthopantomograph as well as casts (Figures 3 and 4). The treatment plan included extraction of deciduous second molar on the left side as well as surgical extraction of the macrodontic premolar on the same side with endodontic treatment and crown for the macrodontic molariform second premolar on the right side followed by a crown on that tooth. This was to be followed by orthodontic correction. Unfortunately the patient did not turn up for the treatment as the patients' parents did not agree to surgical removal of the impacted tooth as that was not giving the child any trouble. The patient was told to come for regular checkup to see the progress of the case and intervene later when patient agrees to treatment.

## 3. Discussion

Nonsyndromic bilateral macrodontia of mandibular second premolars is an extremely rare dental anomaly with very few cases reported to date. Mandibular second premolars show many variations in their morphology; that is to say, the anatomy of this tooth is particularly hard to predict. There can be variations in the cusp wherein we can see one, two, or three cusps on the buccal and lingual side of the tooth [9]. Apart from all these variations which fall within the normal range of variations these teeth can show an extremely rare form of anomaly: molarization. Numerous cases in which mandibular second premolar presents a variety of differences are there in literature including missing teeth, hypodontia, dens in dente, duplication of premolars, and the very rare molarization [5].

Being an extremely rare condition [10], macrodontia of mandibular second premolars has been reported exclusively in children (8–14 years) with only few exceptions [11]. Indeed, disturbances with the eruption of macrodont second premolars and concomitant disruption of developing occlusion or alveolar/gingival enlargement become evident before or between the ages of 11 and 12, when the eruption of mandibular second premolars usually occurs [5]. Thus, any intervention should be completed before maturity, and, in light of previous reports, extraction appears to be the only available intervention [5, 10]. Following extraction, orthodontic treatment should be started in a timely manner due to disturbances in the arch and occlusion after surgical intervention. In our case also extractions were planned followed by orthodontic intervention [12].

The mesiodistal size of tooth 35 (13 mm) was higher than the 7.3 mm for a normal size of second mandibular premolar as reported by others but lower to range between 10.6 and 13.1 mm for macrodontic premolars reported by Dugmore on this way [5]; however, buccolingually, tooth 35 (8 mm) presented similar measures as described by Sicher and Dubrul and Dugmore. Further tooth 45 had measures of mesiodistal width of 12 mm and buccolingual width of 10 mm that corresponds to that given by others [5, 13]. Dental anomalies, including macrodontia, are caused by complex multifactorial interactions including genetic, epigenetic, and environmental factors during the long process of dental development [14, 15]. The patient in our case presented a bilateral macrodontia due to an excessive enlargement of the crown of both mandibular second premolars, as reported in another case. According to the classification of macrodontia, this case corresponds to an isolated macrodontia. It is uncommon to see localized macrodontia alone, because generally it is associated with a syndrome; but our patient and her familial history did not present any other condition or syndrome. The term macrodont molariform premolars has been coined by Dugmore to describe the larger premolars which on being large look like molars. Because these premolars are multitubercular, they have been termed molariform. We have also mentioned these premolars as molariform macrodontic premolars because they are larger in size than other normal premolars and they are multitubercular just like molars.

## 4. Conclusion

It is very important for a dental practitioner to be familiar with macrodontia with regard to not only clinical complications but also its management. Macrodontia also can provide valuable clues in detecting its association with many syndromes and other systemic conditions.

## Figures and Tables

**Figure 4:**